# Draft Genome Sequence of Erythromycin-Resistant *Streptococcus gallolyticus* subsp. *gallolyticus* NTS 31106099 Isolated from a Patient with Infective Endocarditis and Colorectal Cancer

**DOI:** 10.1128/genomeA.00370-15

**Published:** 2015-04-23

**Authors:** Stanimir Kambarev, Clément Caté, Stéphane Corvec, Frédéric Pecorari

**Affiliations:** a Recherche en Oncologie Nucléaire, Centre de Recherche en Cancérologie de Nantes-Angers, INSERM UMR 892, CNRS 6299, Université de Nantes, Nantes, France; b Service de Bactériologie et Hygiène hospitalière, CHU de Nantes, Nantes, France; c Université de Nantes, EA3826 Thérapeutiques Cliniques et Expérimentales des Infections, UFR de Médecine, Nantes, France

## Abstract

*Streptococcus gallolyticus* subsp. *gallolyticus* is known for its close association with infective endocarditis and colorectal cancer in humans. Here, we report the draft genome sequence of highly erythromycin-resistant strain NTS 31106099 isolated from a patient with infective endocarditis and colorectal cancer.

## GENOME ANNOUNCEMENT

*Streptococcus gallolyticus* subsp. *gallolyticus* (formerly *Streptococcus bovis* biotype I) is a common gut commensal in various animals and humans. However, the species is known for its ability to cause different diseases in birds and mammals as well as for its close association with infective endocarditis and colorectal cancer in humans (1–3). Despite the extensive research on this relationship, the underlying virulence features and pathomechanisms remain unclear (4, 5). Recommended antibiotic therapy for streptococcal endocarditis is a combination of penicillin and aminoglycoside. Although penicillin-resistant strains have not yet been isolated, resistances to kanamycin, streptomycin, and erythromycin have been reported and attributed to the presence of the genes *aph*(*3*')-*III*, *ant*(*6*)-*Ia*, and *ermB*, respectively (6–8). Nevertheless, such resistance determinants were not identified in the available genomes of *S. gallolyticus* subsp. *gallolyticus* (9–13). We report the draft genome of highly erythromycin-resistant *S. gallolyticus* subsp. *gallolyticus* NTS 31106099 isolated from a patient with infective endocarditis and colorectal cancer.

*S. gallolyticus* subsp. *gallolyticus* NTS 31106099 was grown overnight at 37°C on Columbia agar supplemented with 5% horse blood (Oxoid, United Kingdom) in an atmosphere of 5% CO_2_. Genomic DNA extraction was accomplished using a DNeasy blood and tissue kit (Qiagen Gmbh, Germany) according to the manufacturer’s recommendation. A sequencing library was prepared using Nextera XT (Illumina, USA) and sequenced using Illumina MiSeq (2 × 300 bp, pair-ends). A total of 10,190, 802 pair-end reads, corresponding to 2.1 Gb was used for *de novo* assembly in SPAdes 2.5.1 (14). Short and low-coverage contigs were filtered out, resulting in a set of 17 contigs between 857 and 583,716 bp with an average coverage of 235×. Annotation was performed by the NCBI Prokaryotic Genome Automatic Annotation Pipeline (PGAAP) (15). Reordering and comparisons were done using Mauve 2.3.1 (16), ACT 8 (17), and BLAST. Acquired antibiotic resistance genes were identified using ResFinder 2.1 (18).

The final assembly has a total length of 2,311,421 bp, an *N*_50_ of 226 kb, and a G+C content of 37.5%. Annotation revealed 2,198 coding sequences (CDS), 59 tRNAs, 38 pseudo genes, 6 rRNAs, and 1 noncoding RNA. Preliminary comparative analysis uncovered a 44.6-kb strain-specific island (JYKU01000013, UG96_07020-UG96_07300) inserted in a putative RNA methyltransferase gene (Gallo_1429 in UCN34 genome [10]). The element was predicted as a putative Tn*916*-like conjugative transposon and designated Tn*6263*, according to Roberts et al. (19). It contains about 50 CDS ( involved in conjugal transfer, regulation, antibiotic resistance [*aph*(*3*')-*III* (UG96_07105), *ant*(*6*)-*Ia* (UG96_07115), and *ermB* (UG96_07135)], and virulence. About 33% of Tn*6263* shows 85% identity to CTn*7* of *Clostridium difficile* (20). Interestingly, about 76% of the element is 99% identical to contig 36 of recently released draft genome of vancomycin-resistant *Enterococcus faecium* VRE3 (JSET01000036.1). Future studies will shed light on the functionality and prevalence of Tn*6263*.

The draft genome of *S. gallolyticus* subsp. *gallolyticus* NTS 31106099 will be used for identification of virulence features associated with colorectal cancer and infective endocarditis.

### Nucleotide sequence accession numbers. 

The draft sequence of *S. gallolyticus* subsp. *gallolyticus* NTS 31106099 studied in this project has been deposited at DDBJ/EMBL/GenBank under the accession no. JYKU00000000. The version described in this paper is JYKU01000000.
